# Poly-L-arginine promotes asthma angiogenesis through induction of FGFBP1 in airway epithelial cells via activation of the mTORC1-STAT3 pathway

**DOI:** 10.1038/s41419-021-04055-2

**Published:** 2021-08-02

**Authors:** Xu Chen, Manli Miao, Meng Zhou, Jie Chen, Dapeng Li, Ling Zhang, Anjiang Sun, Minglong Guan, Zixi Wang, Ping Liu, Shengquan Zhang, Xiaojun Zha, Xiaoyun Fan

**Affiliations:** 1 Department of Geriatric Respiratory and Critical Care, The First Affiliated Hospital of Anhui Medical University, Hefei, China; 2 Anhui Geriatric Institute, Hefei, China; 3 Key Lab of Geriatric Molecular Medicine of Anhui Province, Hefei, China; 4 Department of Biochemistry and Molecular Biology, School of Basic Medicine, Anhui Medical University, Hefei, China

**Keywords:** Cell signalling, Mechanisms of disease, Asthma, Chronic inflammation

## Abstract

Angiogenesis is a key characteristic of asthma airway remodeling. By releasing cationic granule proteins, such as major basic protein (MBP), activated eosinophils play a prominent role in asthma, but the underlying mechanisms are still not fully understood. In this study, we demonstrated that fibroblast growth factor-binding protein 1 (FGFBP1) was dramatically upregulated in airway epithelial cell lines treated by poly-L-arginine (PLA), a mimic of MBP. Elevated FGFBP1 expression was also detected in asthma clinical samples, as well as in ovalbumin (OVA)-induced chronic asthma mouse models. PLA enhanced FGFBP1 expression through activation of the mechanistic target of rapamycin complex 1-signal transducer and activator of transcription 3 (mTORC1-STAT3) signaling pathway. STAT3 transactivated FGFBP1 by directly binding to the promoter of the *FGFBP1* gene. Furthermore, we identified that FGFBP1 secreted by PLA-treated airway epithelial cells served as a proangiogenesis factor. Lastly, we found the mTORC1-STAT3-FGFBP1 signaling pathway was activated in an OVA-induced chronic asthma model with airway remodeling features. Rapamycin treatment alleviated respiratory symptoms and reduced angiogenesis in asthmatic mice. Therefore, activation of the mTORC1-STAT3-FGFBP1 pathway in the airway epithelium contributes to the progress of angiogenesis and should be targeted for the treatment of asthma.

## Introduction

Bronchial asthma is a chronic inflammatory airway disorder that adversely affects the quality of life in both children and adults [1]. The global prevalence of asthma is estimated to be ~334 million cases, and it has been increasing over the past decade [2]. Asthma is characterized by variable airflow limitation and respiratory symptoms such as coughing, wheezing, and chest tightness, accompanied by eosinophilic airway inflammation and airway wall structural changes called airway remodeling [2, 3]. Despite intensive research in recent years, the pathogenetic mechanisms of airway remodeling are still poorly understood and there is no cure or established means of prevention [3].

Airway remodeling is characterized by a persistently altered airway wall structure, including airway wall thickening, mucus hypersecretion, subepithelial fibrosis, angiogenesis, and increased smooth muscle mass [4]. Airway remodeling contributes to fixed airflow obstruction, even causing resistance to inhaled corticosteroids in patients with asthma [2]. Among the multiple postulated mechanisms, an abnormal increase of angiogenesis in the airway tissue and enhanced expression of proangiogenesis factors are critical contributors to airway structural remodeling [5]. Angiogenesis is a pathophysiological process of generating new capillaries, accompanied by increases in vessel density and size, induced by multiple cytokines and chemokines released by inflammatory and structural cells in bronchial asthma [5, 6]. The increased subepithelial vascularity is dilated and prone to vascular leakage and may contribute to increased vascular permeability, thus aggravating airway edema and airway obstruction. Moreover, the newly formed blood vessels promote circulating inflammatory cell recruitment, consequently accelerating the development of inflammation [7]. However, the initiation mechanism of increased angiogenesis has not been extensively investigated and therapeutic antiangiogenesis interventions have received little attention.

The eosinophil is a prominent cell type in stable and exacerbating asthma [8]. Accumulation of activated eosinophils is found in peripheral blood, bronchoalveolar lavage fluid (BALF), bronchial tissue, and induced sputum from patients with asthma [8, 9]. Accumulating evidence has suggested that in addition to contributing to airway inflammation and hyperresponsiveness, eosinophils are postulated to play a critical role in airway remodeling by releasing cytokines, reactive oxygen species, and cationic granule proteins, such as major basic protein (MBP) [5, 10–12]. MBP, the main effector molecule of eosinophils, is elevated in biological fluids from patients with asthma [13, 14]. MBP in lung tissues is highly toxic to the bronchial epithelium owing to disruption of the lipid bilayer membrane, and also owing to induction of airway remodeling through promoting angiogenesis, pulmonary fibrosis, and the goblet cell metaplasia [13–16]. However, the underlying mechanism by which MBP participates in airway remodeling remains unclear.

Poly-l-arginine (PLA) is a synthetic cationic polypeptide that shares the same cationic charges and arginine contents with MBP [17]. PLA is widely used to mimic many of the effects of MBP in vitro [18–20]. Airway epithelial cells are the primary target of MBP [21]. Their function as the first barrier against inhaled pathogens depends on the structural integrity of these cells [22, 23]. Dysregulated functions of the airway epithelium are a central element in the pathogenesis of airway remodeling [5]. In our previous study, we found that PLA synergistically acts with lipopolysaccharide (LPS) to promote human airway epithelial cells to participate in the progression of airway inflammation [24]. We also explored transcriptome profiling of PLA-treated NCI-H292 using RNA-sequencing (RNA-seq) and found 230 differentially expressed genes compared with the control cells [25].

In the current research, we reanalyzed our RNA-seq data and found that fibroblast growth factor-binding protein 1 (FGFBP1), a proangiogenesis factor, was significantly elevated in PLA-treated airway epithelial cell lines, asthma clinical specimens, and ovalbumin (OVA)-induced chronic asthma model. Further experiments indicated that PLA enhanced FGFBP1 expression and secretion through activation of the mechanistic target of rapamycin (mTOR) complex 1 (mTORC1)-signal transducer and activator of transcription 3 (STAT3) signaling pathway. STAT3 transactivated FGFBP1 through directly binding to the promoter of the *FGFBP1* gene. Moreover, PLA induced angiogenesis by stimulating the synthesis and secretion of FGFBP1 in airway epithelial cells. Importantly, rapamycin treatment suppressed the angiogenesis of chronic asthma mice. We suggest that the mTORC1-STAT3-FGFBP1 pathway serves as a promising target for the treatment of asthma.

## Materials and methods

### Reagents, plasmids, and antibodies

PLA, OVA, PD98059, and SB203580 were purchased from Sigma-Aldrich (St. Louis, MO, USA). Rapamycin, S3I-201, and 10058-F4 were obtained from Selleck Chemicals (Houston, TX, USA). Puromycin was purchased from Beijing Solarbio Science & Technology Co., Ltd (Beijing, China). Lipofectamine RNAiMax was purchased from Thermo Fisher Scientific (Waltham, MA, USA). The vector expressing a constitutively activated STAT3 (STAT3C) (pBabe-STAT3C) and control vector (pBabe) have been described previously [26]. pGL3-Basic and pRL-TK plasmid were from Promega (Madison, WI, USA). Packaging vectors (pVSVG and psPAX2) have been described previously [27]. The FGFBP1 antibody was from R&D Systems (Minneapolis, MN, USA; #196519) and Beijing Bo’aosen Biotechnology Co., Ltd (Beijing, China; #MAB1593). mTOR (#9964), regulatory-associated protein of mTOR (Raptor) (#2280), rapamycin-insensitive companion of mTOR (Rictor) (#2114), STAT3 (#9139), phospho-STAT3 (Tyr705) (#9145), S6 ribosomal protein (#2217), phospho-S6 ribosomal protein (Ser235/236) (#4858), phospho-p70 S6 kinase (p-S6K) (Thr389) (#9205), TSC1 (#6935), TSC2 (#4308), p44/42 mitogen-activated protein kinase (MAPK) (ERK1/2) (#4695), phospho-p44/42 MAPK (ERK1/2) (#4370), p38 MAPK (#8690), phospho-p38 MAPK (#4511), c-Myc (#13987), and β-actin (#4970) antibodies were purchased from Cell Signaling Technology (CST, Danvers, MA, USA). CD31 (#MA5-13188) antibody was from Invitrogen (Carlsbad, CA, USA). Secondary antibodies, such as goat anti-rabbit and goat anti-mouse, were acquired from Santa Cruz Technology (Santa Cruz, CA, USA).

### Cell cultures

Human lung mucoepidermoid carcinoma cells (NCI-H292) were obtained from the Shanghai Institute of Life Sciences, Chinese Academy of Sciences (Shanghai, China). Human bronchial epithelial cells (BEAS-2B) and HEK293T cells were from the American Type Culture Collection (Manassas, VA, USA). Human umbilical vein endothelial cells (HUVECs) were obtained from Bioogenetech (Shanghai, China). Tsc1+/+, Tsc1−/−, Tsc2+/+, and Tsc2−/− murine embryonic fibroblasts (MEFs) have been described previously [26]. All cells were cultured in a complete medium supplemented with 10% fetal bovine serum (FBS, Life Technologies, Carlsbad, CA, USA), 100 μg/mL penicillin, and 100 μg/mL streptomycin (Beyotime Biotechnology, Jiangsu, China) in 5% CO_2_ at 37 °C. Cells were tested for mycoplasma infection on a regular basis using TaKaRa PCR Mycoplasma Detection Set (Clontech Laboratories, EUA). NCI-H292 and HUVEC cells were cultured in Roswell Park Memorial Institute (RPMI)-1640 medium (Hyclone, America). BEAS-2B cells, HEK293T cells, Tsc1+/+, Tsc1−/−, Tsc2+/+, and Tsc2−/− MEFs, were cultured in Dulbecco’s modified Eagle’s medium (Hyclone).

### Quantitative real-time PCR (qRT-PCR)

Total RNA for qRT-PCR from treated cells was isolated using TRIzol reagent (Life Technologies) under conditions that inhibit RNAse activity. The first-strand complementary DNA (cDNA) was synthesized using a RevertAid™ First Stand cDNA Synthesis Kit (Fermetas, Waltham, MA, USA), following the manufacturer’s instructions. qRT-PCR analysis for FGFBP1 was performed using Bio-Rad CFX96 (Bio-Rad Laboratories, Hercules, CA), and its transcript was detected by SYBR Premix Ex Taq™ II (TaKaRa, Shiga, Japan). Glyceraldehyde 3-phosphate dehydrogenase served as an internal control. The primer sequences were listed in Supplementary Table S1.

### Western blot analysis

Total proteins from lung tissues and cells were extracted and separated by 10% sodium dodecyl sulfate–polyacrylamide gel electrophoresis and transferred to a PVDF membrane (Millipore, Billerica, MA, USA), and then were blocked in 5% skim milk. Afterward, the membranes were incubated with primary antibodies specific for the target protein overnight at 4 °C. The bands were incubated with horseradish peroxidase-labeled secondary antibodies and then detected by chemiluminescence.

### RNA interference

Small interfering RNA (siRNA) oligonucleotides for mTOR, Raptor, STAT3, and negative control were synthesized by Shanghai GenePharma Company (Shanghai, China). Cells were transfected with siRNAs using Lipofectamine RNAiMax (Invitrogen) according to the product instructions. The siRNA target sequences used are as follows: mTOR, 5′-CCCUGCCUUUGUCAUGCCU-3′; Raptor, 5′-GGACAACGGCCACAAGUAC-3′; STAT3, 5′-CTGGATAACTTCATTAGCA-3′; negative control (NC), 5′-UUCUCCGAACGUGUCACGU-3′.

### Lentivirus infection

NCI-H292 and BEAS-2B cells were transfected with short hairpin RNAs (shRNAs) targeting Raptor, Rictor, STAT3, and FGFBP1 using lentivirus vector GV248 (GenePharma). The target sequences used were as follows: shRaptor, 5′-GGACAACGGCCACAAGUAC-3′; shRictor, 5′-ACUUGUGAAGAAUCGUAUC-3′; shSTAT3^-1^, 5′-GCGTCCAGTTCACTACTAAAG-3′; shSTAT3^-2^, 5′-GCACCTTCCTGCTAAGATTCA-3′; shFGFBP1, 5′-GAGCTCTCTCTGCACATTCTT-3′; shSc, 5′-TTCTCCGAACGTGTCACGT-3′. These recombinant plasmids were co-transfected into HEK293T cells with packing vectors (pVSVG and psPAX2). The supernatants were collected 48–72 h after transfection, and then filtered to infect target cells. The FGFBP1-overexpressing cell lines were generated by lentivirus vector GV492 containing the full-length sequence of FGFBP1 (Genechem, Shanghai, China). The efficiency of lentivirus infection was validated by western blot and qRT-PCR.

### Reporter constructs and dual-luciferase reporter assay

The FGFBP1 promoter was cloned into the *Kpn* I and *Bgl* II sites of the luciferase reporter plasmid pGL3-Basic (Promega). The primer sequences used were as follows: forward, 5′-CGGGGTACCCCGGTGTGGCCAGATTGCAGTG-3′; reverse, 5′-GAAGATCTTCTCCGCTCTAGTAGGGAGTGC-3′. Three potential STAT3-binding sites on the promoter of the *FGFBP1* gene (R1, −752/−742, CTTCTGAAAAT; R2, −1500/−1490, CTTCCCAGAAA; R3, −1694/−1684, GTACCTGGAAA) were mutated using Q5^®^ Site-Directed Mutagenesis Kit (NEB, Ipswich, MA, USA). The primer sequences used were as follows: FGFBP1-mut-R1, forward: 5′-TTTCTTCTTCGACGAATCTATGAAAGGTGAGGTG-3′; reverse: 5′-TTGTTCACTTTTCCTTAATTC-3′; FGFBP1-mut-R2, forward: 5′-GAAGCACTTCTAGTAAAGACCCCGGAGTC-3′; reverse: 5′-TCTGAGCTTTGCCAGGTA-3′; FGFBP1-mut-R3, forward, 5′-GTGTGAGTACACTTAAATGAGGTTGGAAG-3′; reverse, 5′-GCAGACACATAAATACAC-3′. For promoter activity analysis, wild-type or mutant FGFBP1 promoter reporter vector was transfected into HEK293T cells, and pRL-TK (20 ng) was used as an internal reference. The dual-luciferase reporter gene assay system (Promega) was used to detect luciferase activity.

### Chromatin immunoprecipitation (ChIP) assay

A SimpleChIP^®^ Plus Enzymatic Chromatin IP Kit (CST) with a phospho-STAT3 (Tyr705) antibody (CST, #9145) and control rabbit IgG antibody (Abcam, ab171870) were used to identify STAT3-interacting promoter regions according to the manufacturer’s protocol. The purified DNA was analyzed by standard PCR with Hot Start Taq DNA Polymerase (NEB). The primer sequences used were as follows: the putative STAT3-binding site region (PBR) of human FGFBP1, forward: 5′-CAGGAAGGGGAAGGGCCACAAG-3′; reverse: 5′-TGATCCTTGGGAGGCAGGCAGATT-3′; a nonspecific STAT3-binding region (NBR) of human FGFBP1, forward: 5′-GCCCACTATGAGATCCTGAGCAA-3′; reverse: 5′-CACCAACGTCACACTGAACTGCA-3′.

### Preparation of cell-conditioned medium

NCI-H292 or BEAS-2B cells were cultured as described above and stimulated with PLA (0, 10, and 20 μg/mL) for 24 h. Stably transfected NCI-H292 cells were seeded in a 10 cm dish and incubated for 48 h. Next, the medium was removed and cells were washed twice with phosphate-buffered saline. All cells were then cultured in a fresh serum-deprived medium for 24 h. Following the incubation period, the conditioned medium was collected, centrifuged at 2000 r.p.m. at 4 °C for 10 min, and then filtered through a 0.45 μm membrane (Millipore) to remove cell debris. After centrifugation at 12,000 r.p.m. for 20 min, the supernatants were concentrated by ultrafiltration using Amicon Ultra 10K centrifugal filters (Millipore) at 5000 r.p.m. for 20 min. The supernatants were stored at −80 °C until use.

### HUVEC tube formation assay

A total of 150 µL Matrigel (Corning, USA, #354230) was added to a 48-well plate and incubated at 37 °C for 30 min. Then, HUVECs (3.5 × 10^4^) in 300 µL of prepared cell-conditioned medium were added to each well and incubated at 37 °C in 5% CO_2_. After incubation for 12 h, bright-field images were recorded using a microscope and analyzed with WimTube (https://www.wimasis.com/en/WimTube, Wimasis GmbH, Munich, Germany).

### Chicken chorioallantoic membrane (CAM) assay

Fertilized chicken eggs (*n* = 6 chick embryos per group) were incubated at 37 °C and 60% constant humidity [28]. On embryonic developmental day 8 (EDD 8), eggs were windowed with a 1-cm-diameter window above the air chamber. The surface of the dermic sheet on the floor of the air sac was removed to expose the CAM. A sterile gelatin sponge soaked in prepared cell-conditioned medium was applied to the CAM. Conditioned medium (20 μL) was added to the sterile gelatin sponge twice a day. Then, the window was closed with sterile adhesive tape, and the eggs were incubated under standard conditions for 7 days. On EDD 15, following fixation with stationary solution (methanol:acetone, 1:1) for 30 min, the CAM was separated from the eggs and photographed using a digital camera. CAM blood vessels entering the sponges were counted by two observers in a double-blind manner. All the above operations were conducted in a clean environment.

### Enzyme-linked immunosorbent assay

The levels of secreted FGFBP1 in cell culture supernatants and serum samples from clinical subjects were determined using a human FGFBP1 ELISA Kit (R&D Systems) according to the manufacturer’s instructions.

### Chronic asthma mouse model establishment and therapeutic interventions

Five-week-old female C57BL/6 mice were randomly divided into the control, OVA, and rapamycin groups (*n* = 8 mice per group). Mice in the OVA group received intraperitoneal (i.p.) injection of a solution containing 0.5 mg/mL OVA and 5 mg/mL alum on days 0 and 14. From day 21 onward, mice received only 1% OVA intranasally (i.n.) each day for 1 week. Starting on day 28, mice were challenged i.n. with 1% OVA once every other day for 7 weeks. Meanwhile, in the rapamycin group, rapamycin (i.p., 4 mg/kg/day) was continued throughout the challenge period and before the mice received OVA i.n. until day 78. Untreated control groups included mice challenged with saline alone on the same schedule (Fig. 8A). On day 78, the mice were killed and samples were collected for further analysis, as described previously [29]. The studies were approved by the Experimental Animal Ethical Committee of Anhui Medical University (LLSC-20200806).

### Histology and immunohistochemistry (IHC)

The lung tissues of mice were fixed in 4% paraformaldehyde. All tissue specimens were then paraffin-embedded, and 4-μm-thick histology sections were prepared. For histological analyses, hematoxylin and eosin (H&E) staining was performed according to standard procedures to examine the bronchial inflammatory infiltration and the inflammatory score was evaluated as described previously [29]. Masson’s trichrome staining was used to detect collagen fibers. The perimeter of the basement membrane (Pbm) and total area of the airway wall (Wat) were measured by Image-Pro Plus 6.0 and the ratios of Wat to Pbm (Wat/Pbm) were evaluated. To examine the protein expression in the tissues, antibodies against phospho-S6 ribosomal protein (Ser235/236) (1:400, CST, #4858), phospho-STAT3 (Tyr705) (1:200, CST, #9145), FGFBP1 (1:200, Beijing Bo’aosen, #MAB1593), and CD31 (1:100, Invitrogen, #MA5-13188) were used as primary antibodies for IHC.

### Case selection and clinical samples obtain

A total of 28 volunteers were recruited for this study, including patients with asthma (*n* = 13) and healthy controls without asthma (*n* = 15). Human participants in this study signed informed consent to participate in this research and underwent blood sampling. The concentration of FGFBP1 and eosinophil count for all patients and controls were measured. This project was approved by the Ethical and Scientific Committee of the First Affiliated Hospital of Anhui Medical University (20200814).

### Statistical analysis

All statistical analyses were performed using GraphPad Prism (version 6) and IBM SPSS version 23.0 (IBM Corp., Armonk, NY, USA). The sample sizes of all experiments were selected based on our experience and previous studies [27, 29]. Animals were not excluded from the analysis as outliers. Prior to making comparisons across values, the normality of distributions was tested using the Kolmogorov–Smirnov test. The variance was similar between groups. Pearson’s correlation analysis was used to evaluate the association between FGFBP1 expression and eosinophil count. Differences in the distribution of individual features were analyzed using the *χ*^2^ test for categorical variables and the Mann–Whitney *U* test for continuous variables. All reported *P* values are two-tailed, with values <0.05 indicating statistical significance. All data are representative of at least three separate experiments.

## Results

### PLA elevates the expression of FGFBP1 in airway epithelial cells

**Fig. 1:**
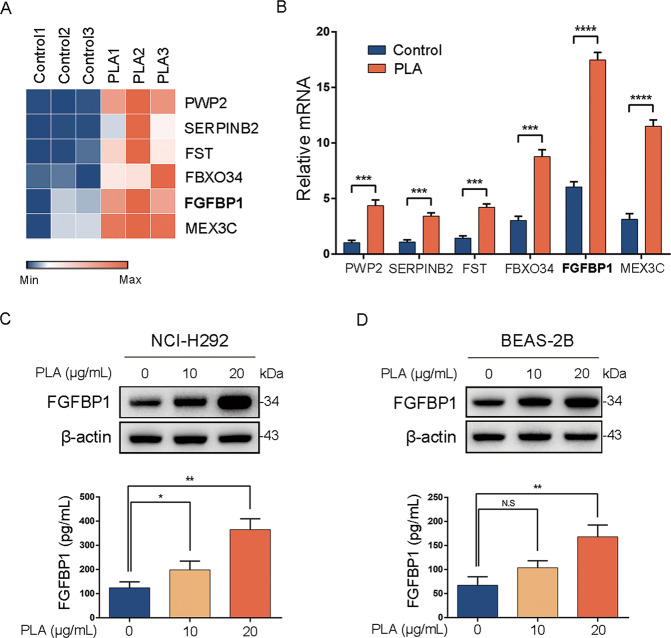
PLA elevates the expression of FGFBP1 in airway epithelial cells. **A** Heatmap showing the top six differentially expressed genes in PLA-treated (PLA) or untreated (Control) NCI-H292 cells based on RNA-sequencing results. **B** NCI-H292 cells were treated with or without PLA (20 μg/mL, 24 h) and examined for the expression of *PWP2*, *SREPINB2*, *FST*, *FBXO34*, *FGFBP1*, and *MEX3C* (normalized to GAPDH) by qRT-PCR. **C**, **D** Human airway epithelium cell lines, NCI-H292 and BEAS-2B, were treated with PLA (0, 10, and 20 μg/mL) for 24 h. Cell lysates were subjected to immunoblotting (upper panels) and FGFBP1 levels in the cell supernatants were detected using ELISA (lower panels). Results are shown as mean ± SD from three independent experiments. *P < 0.05; **P < 0.01; ***P < 0.001; ****P < 0.0001; NS not significant.

To explore the influence of PLA, a mimic of MBP, on airway epithelial cells, we reanalyzed our RNA-seq data of PLA-treated or untreated NCI-H292 cells [25]. Here, we list the top six upregulated genes with PLA treatment, including *PWP2*, *SREPINB2*, *FST*, *FBXO34*, *FGFBP1*, and *MEX3C* (Fig. 1A). qRT-PCR analysis was performed to examine the expressions of these genes in response to PLA in NCI-H292 cells. The results showed that messenger RNA (mRNA) expressions of these genes were indeed increased by PLA treatment (Fig. 1B). Among the six PLA-responsive genes, *FGFBP1* encodes a secreted carrier protein, which functions with fibroblast growth factor-2 (FGF-2) to activate angiogenesis and tissue repair [30]. Angiogenesis is an important characteristic of airway remodeling in asthma. Therefore, we focused on FGFBP1 in this study. Consistent with qRT-PCR data, the protein expression of FGFBP1 was significantly overexpressed in NCI-H292 cells treated with PLA at 10 or 20 μg/mL by western blot (Fig. 1C, upper panel). Moreover, we determined that FGFBP1 levels were also significantly higher in the supernatants of PLA-treated NCI-H292 cells than in the control cells using ELISA (Fig. 1C, lower panel). Similarly, treated human bronchial epithelium BEAS-2B cells with PLA also led to dramatic expression and secretion of FGFBP1 (Fig. 1D). Collectively, these data indicated that PLA could lead to a marked upregulation of FGFBP1 expression and secretion in airway epithelial cell lines.

### FGFBP1 correlates to eosinophil count in asthmatic peripheral blood

**Fig. 2:**
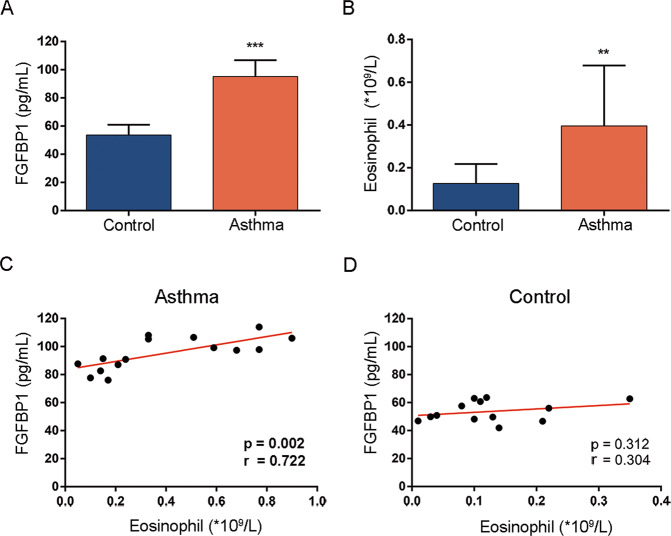
FGFBP1 correlates to eosinophil count in asthmatic peripheral blood. **A** FGFBP1 protein levels in the peripheral blood of human subjects were quantified by ELISA. **B** Peripheral blood eosinophil counts of patients with asthma (*n* = 13) and healthy controls without asthma (*n* = 15) were obtained using an automated cell counter. **C**, **D** Pearson’s correlation scatter plot of the concentration of FGFBP1 and peripheral blood eosinophil count in asthmatic peripheral blood (*r* = 0.722, *P* = 0.002; **C**) and in participants without asthma (*r* = 0.304, *P* = 0.312; **D**). Results are shown as mean ± SD. **P < 0.01; ***P < 0.001.

Activated eosinophils in the asthmatic airway wall contribute to airway inflammation and airway remodeling by releasing highly charged cytotoxic granular proteins, such as MBP [13]. Given that PLA is widely used to mimic MBP in vitro [13, 18] and PLA-induced FGFBP1 expression in airway epithelial cells (Fig. 1C, D), we speculated that FGFBP1 is overexpressed in patients with asthma. Therefore, we analyzed the FGFBP1 concentration in peripheral blood samples from 28 participants (13 with asthma and 15 healthy controls without asthma) using ELISA. The characteristics of participants are listed in Supplementary Table S2. ELISA demonstrated that the concentration of FGFBP1 was higher in asthmatic peripheral blood than in healthy controls (Fig. 2A). Moreover, patients with asthma had a markedly increased eosinophil count compared with healthy controls (Fig. 2B). We next analyzed the correlation between FGFBP1 concentration and eosinophil count in patients with asthma and controls, respectively (Fig. 2C, D). The results showed that FGFBP1 expression in patients with asthma was strongly correlated with eosinophil count (*r* = 0.722, *P* = 0.002). However, there was no significant correlation between eosinophil count and FGFBP1 in participants without asthma (*r* = 0.304, *P* = 0.312). Taken together, these data indicated that FGFBP1 was higher in patients with asthma than in controls, and its concentration in asthmatic peripheral blood was closely related to the activated eosinophils.

### PLA upregulates FGFBP1 expression through activation of mTORC1

**Fig. 3:**
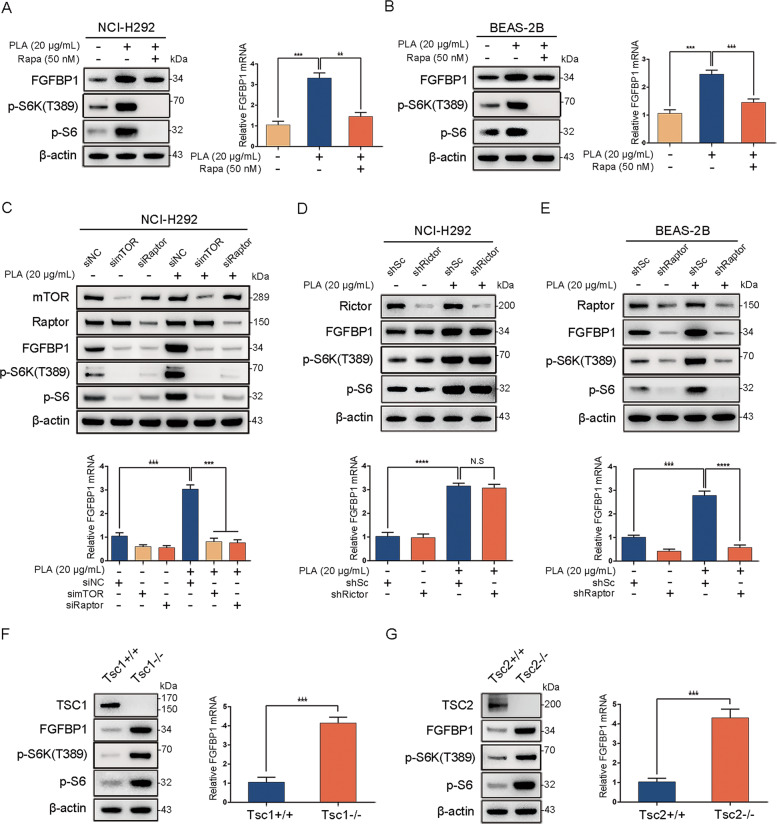
PLA upregulates FGFBP1 expression through activation of mTORC1. **A**, **B** NCI-H292 (**A**) and BEAS-2B (**B**) cells were pretreated with PLA (20 μg/mL, 12 h) before rapamycin (50 nM, 12 h) treatment. **C** NCI-H292 cells were transfected with siRNA targeting mTOR (simTOR), Raptor (siRaptor), or the control (siNC) for 48 h before PLA (20 μg/mL, 12 h) treatment. **D** NCI-H292/shRictor or NCI-H292/shSc cells were treated with PLA (20 μg/mL) for 24 h. **E** BEAS-2B/shRaptor or BEAS-2B/shSc cells were treated with PLA (20 μg/mL) for 24 h. **F** Tsc1+/+ and Tsc1−/− MEFs. **G** Tsc2+/+ and Tsc2−/− MEFs. **A**–**G** All cell lysates were collected for western blot with the indicated antibodies (**A**, **B**, **F**, and **G**, left panels and **C**–**E**, upper panels) and FGFBP1 mRNA was detected by qRT-PCR (**A**, **B**, **F**, and **G**, right panels and **C**–**E**, lower panels). Results are shown as mean ± SD from three independent experiments. **P < 0.01; ***P < 0.001; ****P < 0.0001; NS not significant.

mTOR serves as a cellular process regulator center in cell cycle progression, cell growth, differentiation, survival, and angiogenesis [31–33]. Accumulating evidence also shows that mTOR plays a critical role in eosinophil-associated diseases [34–37]. Therefore, we next examined whether the activity of mTOR is activated by PLA. Immunoblot analysis revealed that PLA did upregulate the phosphorylation levels of p-S6K and ribosomal S6 (p-S6), established indicators of mTORC1 activity, in both NCI-H292 and BEAS-2B cells (Supplementary Fig. S1). To explore whether the upregulated expression of FGFBP1 in airway epithelium cells treated with PLA was directly owing to mTORC1 activation, we first treated these cells with rapamycin, an inhibitor of mTORC1. As shown in Fig. 3A, in NCI-H292 cells, inhibition of mTORC1 with rapamycin led to the suppression in both protein and mRNA levels of FGFBP1 induced by PLA. Consistently, rapamycin treatment attenuated FGFBP1 expression elevated by PLA in BEAS-2B cells (Fig. 3B). In cells, mTOR exists in two complexes with different components such as Raptor (named mTORC1) and Rictor (named mTORC2) [38]. To further verify that mTORC1 was involved in the PLA enhancement of FGFBP1, NCI-H292 cells were transfected with mTOR and Raptor siRNAs before PLA treatment. Both mTOR- and Raptor-knockdown cells displayed lower FGFBP1 expression compared with the control cells in response to PLA treatment (Fig. 3C), while depletion of Rictor with Rictor shRNA had little effect on the expression of FGFBP1 in NCI-H292 cells (Fig. 3D). Moreover, knockdown of Raptor by transfecting Raptor shRNA expression lentiviruses to BEAS-2B cells also led to significantly repressed FGFBP1 (Fig. 3E). The TSC1/TSC2 complex is the main upstream regulator of mTOR, and hyperactivated mTOR, caused by the loss of TSC1 or TSC2, leads to uncontrolled cell proliferation and tumor growth [31]. Thus, Tsc1−/− and Tsc2−/− MEFs and their corresponding MEFs are widely used in the study of mTOR signaling [39–41]. Here, we performed immunoblotting analysis and qRT-PCR on the two pairs of MEFs. In line with the above findings, mTOR-activated cells had greater FGFBP1 expression compared with the control cells (Fig. 3F, G). All these results suggested that PLA enhanced FGFBP1 expression through mTORC1 at the levels of both protein and mRNA.

### mTORC1 enhances FGFBP1 expression through activation of STAT3

**Fig. 4:**
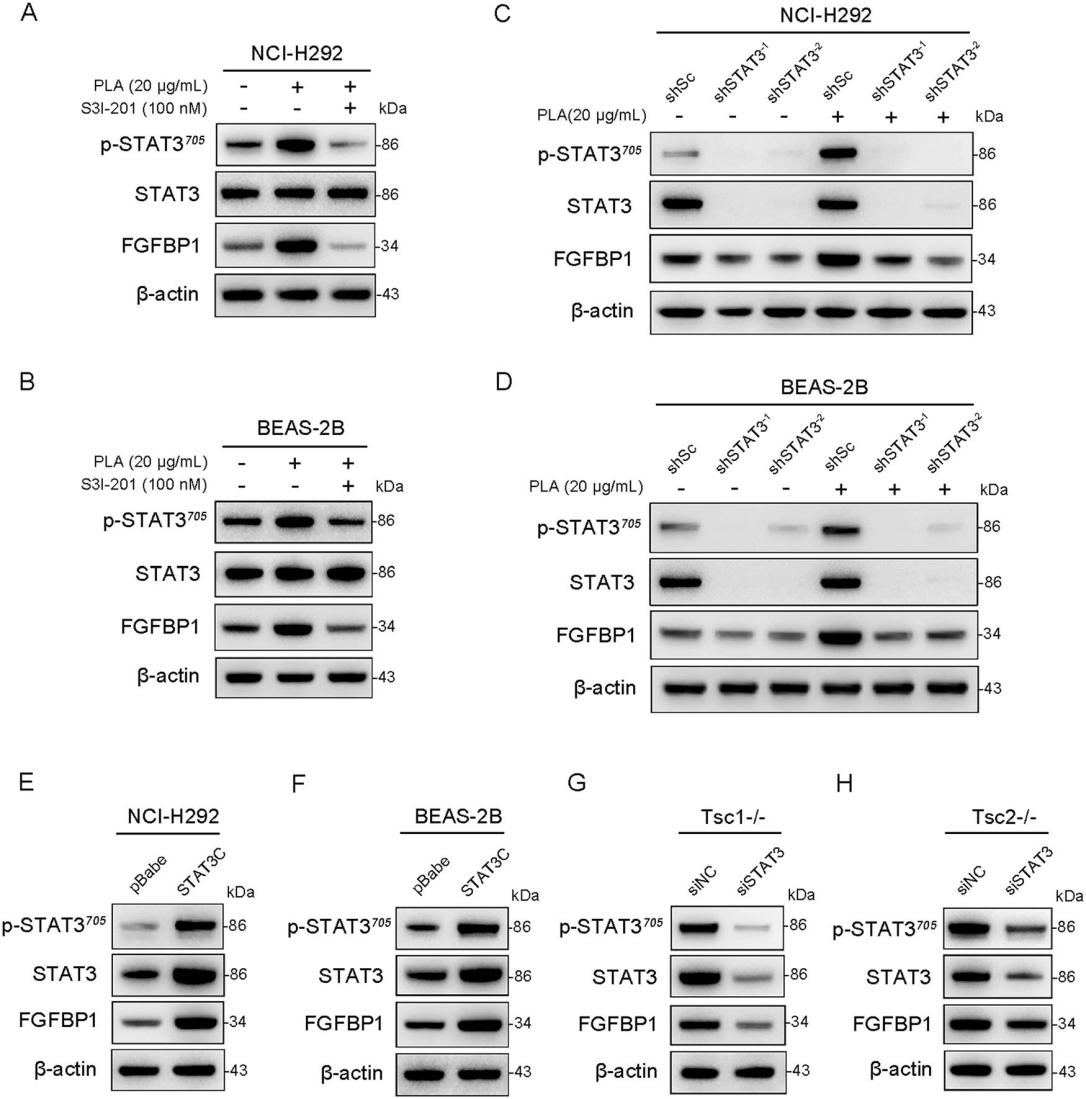
mTORC1 enhances FGFBP1 expression through activation of STAT3. **A**, **B** NCI-H292 (**A**) and BEAS-2B (**B**) cells were pretreated with PLA (20 μg/mL, 12 h) before the addition of S3I-201 (100 nM) for 12 h. **C**, **D** NCI-H292 (**C**) and BEAS-2B (**D**) cells, stably expressed lentivirus shSTAT3^-1^, shSTAT3^-2^, or shSc, were treated with or without PLA (20 μg/mL, 24 h). **E**, **F** NCI-H292 (**E**) and BEAS-2B (**F**) cells were transfected with pBabe-STAT3C, a constitutively active STAT3 mutant, or the control vector pBabe. **G**, **H** siRNAs against STAT3 (siSTAT3) or the control (siNC) were transfected into Tsc1−/− MEFs (**G**) and Tsc2−/− MEFs (**H**). **A**–**H** Cell lysates of the indicated cells were harvested for immunoblot analysis with the indicated antibodies. All experiments were performed in triplicate.

STAT3, a well-known downstream effector of mTORC1, plays a critical role in angiogenesis [42, 43]. Given that PLA treatment led to the activation of mTORC1 as well as STAT3 (Supplementary Fig. S1) in both NCI-H292 and BEAS-2B cells, we hypothesized that PLA increases the expression of FGFBP1 through activation of the mTORC1-STAT3 signaling pathway. As shown in Fig. 4A, B, inhibition of STAT3 activity using S3I-201, a specific STAT3 inhibitor, dramatically decreased PLA-induced FGFBP1 expression in NCI-H292 and BEAS-2B cells. Similarly, knocking down STAT3 using two independent shRNAs (shSTAT3^-1^ and shSTAT3^-2^) markedly attenuated the stimulatory effect of PLA on FGFBP1 expression (Fig. 4C, D). To further investigate the relationship between STAT3 and FGFBP1, STAT3C was introduced into airway epithelial cells. The results showed that the expression of FGFBP1 was substantially elevated in STAT3C-transfected cells compared with the control (Fig. 4E, F). We also detected FGFBP1 expression in STAT3 siRNA-transfected Tsc1−/− or Tsc2−/− MEFs and found that depletion of STAT3 significantly reduced FGFBP1 expression (Fig. 4G, H). Taken together, these results indicated that mTORC1 mediated the stimulatory effects of PLA on FGFBP1 expression through activation of STAT3.

### STAT3 upregulates FGFBP1 at the transcriptional level

**Fig. 5:**
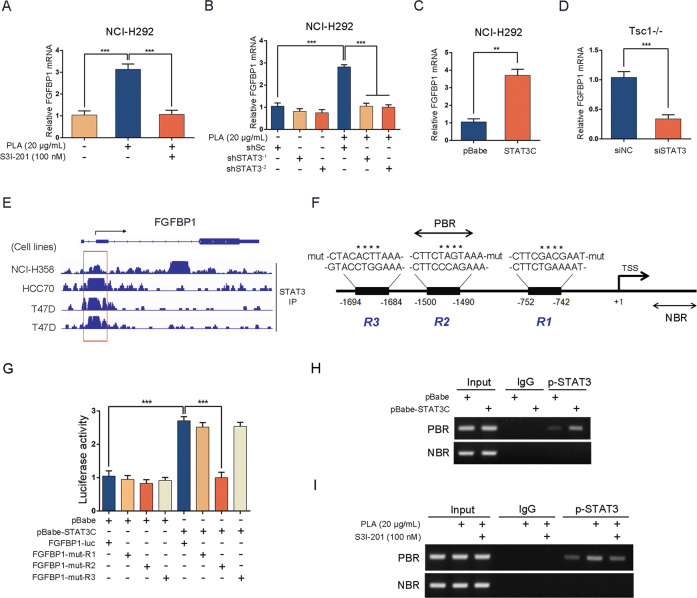
STAT3 upregulates FGFBP1 at the transcriptional level. **A**, **I** NCI-H292 cells were pretreated with PLA (20 μg/mL, 12 h), followed by S3I-201 (100 nM, 12 h) treatment. **B** NCI-H292/shSTAT3^-1^, NCI-H292/shSTAT3^-2^, or NCI-H292/shSc cells were treated with or without PLA (20 μg/mL) for 24 h. **C**, **H** NCI-H292 cells were transfected with pBabe-STAT3C or pBabe. **D** siSTAT3 or siNC were transfected into Tsc1−/− MEFs. **A**–**D** The mRNA level of FGFBP1 was detected by qRT-PCR. **E** ChIP-Atlas analysis of STAT3 ChIP-Seq experiments performed in human lung cancer cell line (NCI-H358) and breast cancer cell lines (HCC70 and T47D). **F** Schematic representation of the putative wild-type and three mutated (mut) STAT3-binding sites in the promoter of human *FGFBP1* gene from Jaspar transcription profile database (http://jaspar.genereg.net). The mutated bases of putative STAT3-binding sites were denoted by *. **G** HEK293T cells were co-transfected with FGFBP1-luc, FGFBP1-mut-R1, FGFBP1-mut-R2, or FGFBP1-mut-R3 together with pBabe-STAT3C or pBabe. Relative luciferase activity was detected 24 h after transfection. **H**, **I** Cells were subjected to ChIP assay. Normal rabbit IgG antibody served as the negative control. PCR was performed to amplify regions surrounding the putative STAT3-binding region (PBR) and a nonspecific STAT3-binding region (NBR). All experiments were performed in triplicate. Results are shown as mean ± SD. **P < 0.01; ***P < 0.001.

To further explore the underlying mechanisms of upregulated FGFBP1 by STAT3, total RNA was isolated from S3I-201-treated NCI-H292 cells. qRT-PCR was performed to detect the mRNA level of FGFBP1. As shown in Fig. 5A, the PLA-induced expression of FGFBP1 mRNA was significantly attenuated in the presence of S3I-201. Consistently, knockdown of STAT3 using shSTAT3^-1^ or shSTAT3^-2^ also led to a marked reduction in the mRNA level of FGFBP1 induced by PLA (Fig. 5B). In contrast, ectopic expression of a STAT3C enhanced FGFBP1 mRNA in NCI-H292 cells (Fig. 5C). In addition, knockdown of STAT3 by transfecting STAT3 siRNA into Tsc1−/− MEFs led to the reduction of FGFBP1 mRNA (Fig. 5D). To investigate whether STAT3 directly promotes transcription of FGFBP1, we explored the STAT3-binding site(s) around the transcription initiation regions of the *FGFBP1* gene using ChIP-Atlas, an extensive database of publicly available ChIP-sequencing experiments (http://chip-atlas.org). As shown in Fig. 5E, STAT3 bound to the 5′ region of the *FGFBP1* gene in a human lung cancer cell line (NCI-H358) and breast cancer cells (HCC70 and T47D). Moreover, we identified three putative STAT3-binding sites (R1, −752/−742, CTTCTGAAAAT; R2, −1500/−1490, CTTCCCAGAAA; and R3, −1694/−1684, GTACCTGGAAA) in a 2.0 kb region upstream to the transcription starting site (TSS) of the *FGFBP1* gene using the Jaspar transcription profile database (http://jaspar.genereg.net) (Fig. 5F and Supplementary Table S3). The fragment from −1959 to +28 was fused to the promoter-lacking firefly luciferase gene to generate an FGFBP1-luc reporter. The above three putative binding sites were mutated to generate an FGFBP1-mut-R1 reporter, FGFBP1-mut-R2 reporter, and FGFBP1-mut-R3 reporter, respectively. Next, the recombinant reporter plasmid was then co-transfected into HEK293T cells together with pBabe-STAT3C or the empty vector (pBabe), as well as the internal control plasmid pRL-TK. As shown in Fig. 5G, increased luciferase activity in STAT3-overexpressed cells suggested significant transcriptional activation of FGFBP1. Furthermore, the transcriptional activity was notably reduced when the putative STAT3-binding site R2 was mutated, whereas neither mutated R1 nor R3 was altered. These results indicated that R2 (−1500/−1490, CTTCCCAGAAA) is likely to be the binding site of STAT3. Furthermore, ChIP analysis further revealed that the binding of STAT3 to this putative site was enhanced by transfecting STAT3C into NCI-H292 cells (Fig. 5H). Also, the binding of STAT3 to this site was markedly stronger in PLA-treated NCI-H292 cells than in the control cells and was attenuated by suppression of STAT3 with S3I-201 (Fig. 5I). Collectively, these data provide powerful support for the hypothesis that STAT3 promotes the transcription of FGFBP1 through directly binding to the promoter of the *FGFBP1* gene.

### PLA promotes angiogenesis in vitro and in vivo

**Fig. 6:**
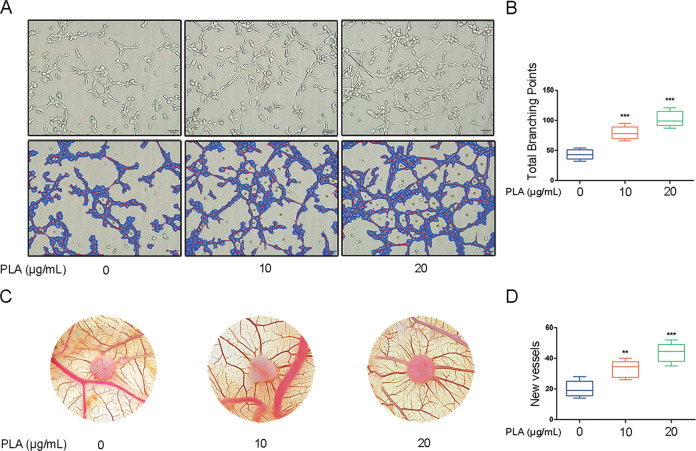
PLA promotes angiogenesis in vitro and in vivo. **A**–**D** NCI-H292 cells were treated with PLA (0, 10, and 20 μg/mL) for 24 h and the cell-conditioned media were collected for tube formation assay (**A**, **B**) and CAM assay (**C**, **D**). For tube formation assay (**A**, **B**), HUVECs (3.5 × 10^4^) were mixed with prepared conditioned media and incubated for 12 h on Matrigel. **A** Representative micrographs of tube formation assay. **B** Quantifications of total branching points per microscopic field from three independent experiments were analyzed by WimTube. For CAM assay (**C**, **D**), on EDD 8, sterile gelatin sponges were applied to the CAMs and loaded with 20 μL prepared conditioned medium twice per day. **C** Representative images of the CAM blood vessels. **D** CAM blood vessels entering the sponges were counted by two observers in a double-blind manner. *n* = 6 chick embryos per group. Results are shown as mean ± SD. **P < 0.01; ***P < 0.001.

Given that FGFBP1 is a well-established proangiogenesis factor and PLA stimulates FGFBP1 expression in airway epithelium cells, we speculated that PLA could promote angiogenesis. Cell-conditioned media collected from PLA-treated NCI-H292 cells were assayed for angiogenic activity. HUVECs were resuspended in prepared conditioned medium and plated on Matrigel-coated plates. As shown in Fig. 6A, B, HUVEC tube formation was increased by adding the prepared PLA-treated NCI-H292 supernatants, which led to much higher total branching points as quantified by WimTube (https://www.wimasis.com/en/WimTube). Furthermore, using the CAM system, we examined the effect of PLA on angiogenesis in vivo and evaluated new vessel formation. Consistent with our in vitro results above, the supernatant of PLA-stimulated NCI-H292 cells induced a stronger angiogenic response in a chicken embryo CAM assay than in the control cells. Moreover, the angiogenic responses to the gelatin sponge in the PLA-treated group were surrounded by allantoic vessels that developed radially toward the implant in a “spoked wheel” pattern; the CAM incubated without PLA stimulation showed no vascular reaction around the sponge (Fig. 6C, D). To conclude, our results implied that PLA exhibited proangiogenesis functions both in vitro and in vivo.

### PLA promotes angiogenesis through FGFBP1

**Fig. 7:**
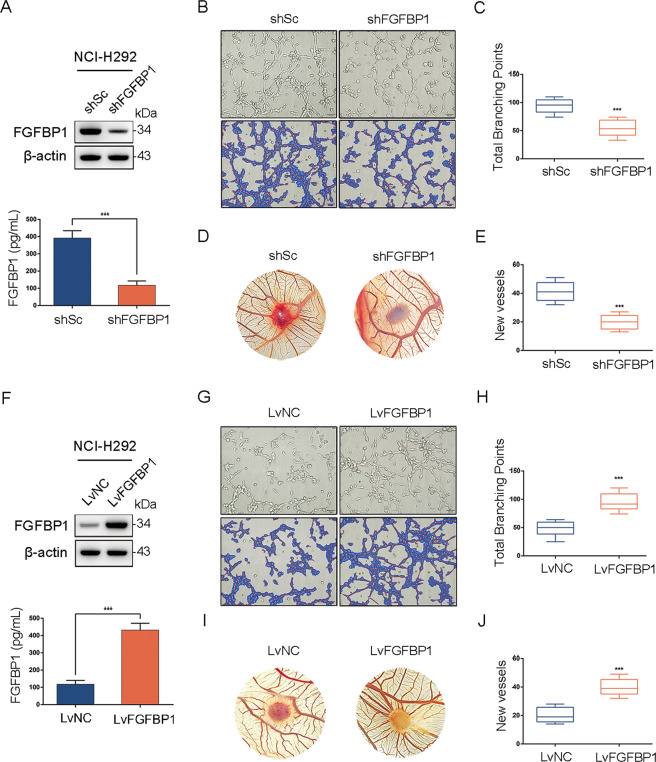
PLA promotes angiogenesis through FGFBP1. **A** NCI-H292 cells, stably expressed shRNA targeting FGFBP1 (shFGFBP1) or a control shRNA (shSc), were treated with 20 μg/mL PLA for 24 h. Western blot (upper panel) and ELISA (lower panel) were performed to detect the protein levels of FGFBP1 in cell lysates and supernatants, respectively. **B**–**E** NCI-H292/shFGFBP1 and NCI-H292/shSc cells were treated with PLA (20 μg/mL) for 24 h and the cell-conditioned media were collected for tube formation assay (**B**, **C**) and CAM assay (**D**, **E**). **B**, **D** Representative micrographs of tube formation assay (**B**) and CAM assay (**D**). **C**, **E** Quantitations of total branching points of tube formation assay (**C**) and new vessels of CAM assay (**E**). **F** NCI-H292 cells infected with lentivirus harboring a vector encoding FGFBP1 (LvFGFBP1) or the empty vector (LvNC). Western blot (upper panel) and ELISA (lower panel) were performed to detect the protein levels of FGFBP1 in cell lysates and supernatants, respectively. (**G**–**J**) The cell-conditioned media of NCI-H292/LvFGFBP1 and NCI-H292/LvNC cells were collected for tube formation assay (**G**, **H**) and CAM assay (**I**, **J**). **G**, **I** Representative micrographs of tube formation assay (**G**) and CAM assay (**I**). **H**, **J** Quantitation of total branching points of tube formation assay (**H**) and new vessels of CAM assay (**J**). *n* = 6 chick embryos per group. Results are shown as mean ± SD. ***P < 0.001.

Considering that FGFBP1 in the epithelium was upregulated by PLA treatment (Fig. 1), we wondered whether this is owing to upregulated FGFBP1 that mediates the proangiogenesis effects of PLA. First, a lentiviral vector expressing shRNA targeting FGFBP1 (shFGFBP1) was transfected to NCI-H292 cells. Subsequent western blot and ELISA affirmed that shFGFBP1 led to a dramatic decrease of PLA-induced FGFBP1 expression compared with the control (shSc) (Fig. 7A). As shown in Fig. 7B, C, depletion of FGFBP1 led to a marked decrease in PLA-induced tube formation compared with the control. Consistently, vascular clustering with shFGFBP1 cell supernatant in CAM systems showed similar results (Fig. 7D, E). To further explore the angiogenic effects of FGFBP1, we overexpressed FGFBP1 in NCI-H292 cells using a lentiviral vector, verified with immunoblot analysis and ELISA (Fig. 7F). The ectopic expression of FGFBP1 dramatically exerted promotional effects on angiogenesis, like PLA (Fig. 7G–J). Altogether, these data showed that FGFBP1 generated by NCI-H292 cells was essential in PLA-induced angiogenesis.

### mTORC1-STAT3-FGFBP1 pathway regulates angiogenesis in an OVA-induced chronic asthma model

**Fig. 8:**
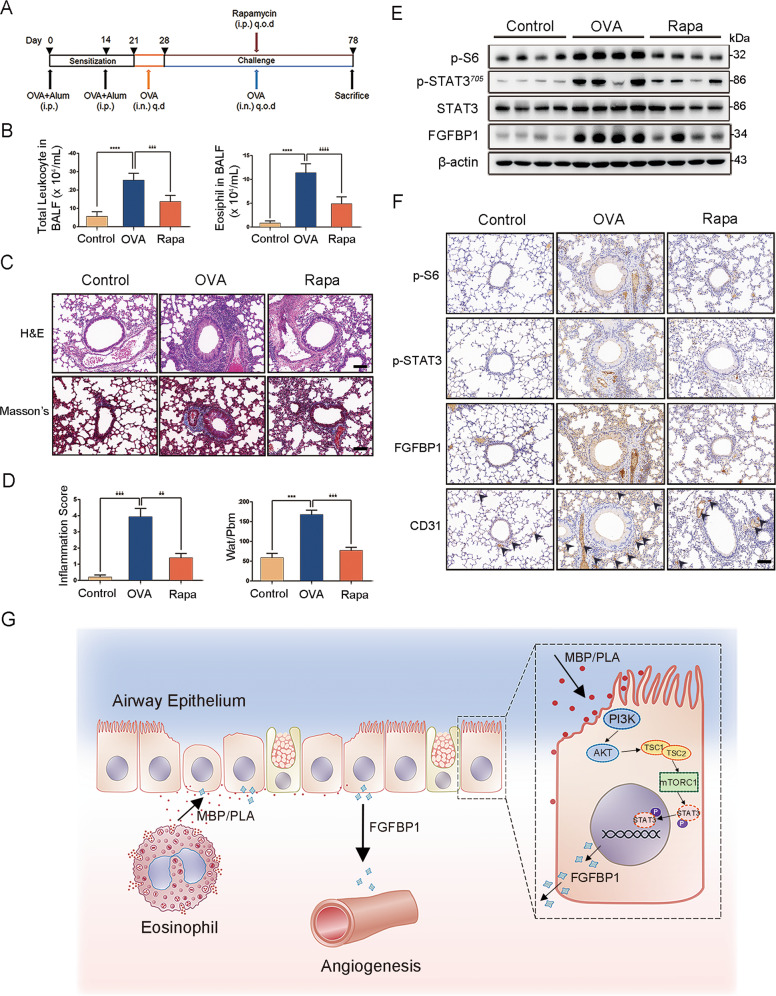
mTORC1-STAT3-FGFBP1 pathway regulates angiogenesis in an OVA-induced chronic asthma model. **A** The timeline to establish OVA-induced chronic asthma models and therapeutic interventions (see the “Materials and methods” section for details). The mice were divided into three groups: the control group (Control), OVA-induced chronic asthma group (OVA), and rapamycin-treated group (Rapa). **B** The total number of leukocytes and eosinophil count in the BALF were counted using Wright–Giemsa staining. **C** Representative photographs of H&E staining (upper panels) and Masson’s staining (lower panels) of lung tissues (scale bar: 100 μm). **D** Inflammation score (left panels) and Wat/Pbm (right panels) of lung tissues. **E** Immunoblotting of the indicated proteins in lung tissues. **F** IHC analysis of the indicated proteins in lung tissues (scale bar: 100 μm). *n* = 8 mice per group. Results are shown as mean ± SD. **P < 0.01; ***P < 0.001; ****P < 0.0001. **G** Schematic illustration of activated mTORC1-STAT3-FGFBP1 signaling pathway promotes angiogenesis in airway epithelial cells.

To assess possible links between the mTORC1-STAT3-FGFBP1 pathway and airway remodeling in vivo, we established a mouse model of OVA-induced chronic asthma. To induce chronic asthma, C57BL/6 mice received OVA/aluminum for sensitization. During the challenge stage, they were pretreated with rapamycin or saline 1 h before the OVA challenge (Fig. 8A). Mice in the asthma group displayed wheezing, shortness of breath, and dyspnea. However, no such symptoms were observed in mice receiving rapamycin treatment. The total number of leukocytes and eosinophils in the BALF of OVA-induced mice was highly elevated in comparison with those of the control mice (Fig. 8B). Rapamycin treatment reduced the total number of leukocytes and eosinophils in BALF, indicating that rapamycin could alleviate airway inflammation development (Fig. 8B). As shown in Fig. 8C (upper panel) and 8D (left panel), H&E staining revealed that OVA mice exhibited greater inflammation infiltration and higher inflammation score in lung tissues than in control mice, whereas the rapamycin-treated group mice dramatically repressed these effects. Moreover, as shown in Fig. 8C (lower panel) and 8D (right panel), Masson’s trichrome staining revealed that collagenous fiber deposition (indicated by blue staining) was increased in OVA-challenged group, which was accompanied by high Wat/Pbm values, whereas the thickening was reduced after rapamycin treatment. Further, immunoblotting analysis and IHC staining were performed to detect the mTORC1-STAT3-FGFBP1 signaling pathway successively. As shown in Fig. 8E, the mice with asthma consistently exhibited higher levels of p-S6, p-STAT3, and FGFBP1 protein than in the control group. Their expressions were reduced after rapamycin treatment. Consistently, similar results were observed in IHC staining (Fig. 8F). We also performed CD31 staining, a marker for vascular endothelial cells, in mice lung tissues. CD31 expression in the blood vessels of lung tissues was consistent with the expression of p-S6, p-STAT3, and FGFBP1. As shown in Fig. 8F, CD31-positive staining of the vessel lumen of OVA-induced mice was markedly increased in comparison with control mice and could be repressed with rapamycin treatment. These data indicated that the mTORC1-STAT3-FGFBP1 signaling pathway plays a crucial role in angiogenesis in chronic asthma, and rapamycin treatment can block the progression of angiogenesis in the airway wall.

## Discussion

Increased neovascularization in the asthmatic airway alters the inflamed microenvironment of the bronchial tissue by recruiting more infiltrating inflammatory cells, which results in accelerated deterioration of lung function [5, 6]. Emerging evidence indicates that eosinophils contribute to angiogenesis in airway remodeling that occurs in asthma [7, 44, 45]. For example, one group reported that the expression of angiogenic factors, including vascular endothelial growth factor, FGF-2, and angiogenin, was markedly elevated, accompanied by an increased number of eosinophils, new vessels, and the blood vessels in the submucosa [46]. Another group showed that eosinophil sonicated from peripheral blood of patients with asthma displayed an enhanced capacity of endothelial cell proliferation and inducement of a strong angiogenic response in the aortic rings [45]. In the present study, we showed that PLA, a mimic of MBP (released by eosinophils), promoted angiogenesis through induction of FGFBP1 in airway epithelial cells. FGFBP1 is a secreted carrier protein that binds and activates FGF-2 as an extracellular chaperone in a reversible and non-covalent manner. This binding subsequently allows FGF-2 to be released from the extracellular matrix and bind to FGF receptors, thereby activating angiogenesis and tissue repair [30, 47–49]. Interestingly, Puxeddu et al. reported that by directly adding PLA in a chicken embryo CAM assay, PLA did not induce any angiogenic response [15]. Our current findings may help to explain this phenomenon. PLA is likely to be an indirect angiogenic factor through stimulation of epithelium cells to synthesize and release FGFBP1. Moreover, we found elevated FGFBP1 expression in asthma clinical specimens and an OVA-induced animal model of asthma. Consistent with our findings, Singhania et al. reported that FGFBP1 was upregulated in both central and peripheral airways from patients with severe asthma in comparison with healthy volunteers using RNA-seq [50]. Thus, it is likely that FGFBP1 is a potential therapeutic target of airway remodeling in asthma.

As FGFBP1 is crucial in neovascularization, the upstream regulatory mechanism of FGFBP1 expression has aroused increasing attention [51–53]. Several signaling cascades have been reported to take part in the regulation of FGFBP1 expression. For example, Cottarelli et al. showed that Wnt/β-catenin signaling promoted FGFBP1 expression in the development of the blood–brain barrier [51]. It has been reported that FGFBP1 expression is induced through the RAS-PKC signal transduction pathway in skin carcinogenesis [52]. In addition, ERK and p38 MAPK activation has been suggested to be involved in the upregulation of FGFBP1 in the cervical squamous cell carcinoma cell line ME-180 [53]. In fact, we previously found that PLA synergized, via ERK and p38 MAPK signaling, with LPS to promote the release of interleukin-6 (IL-6) and IL-8 release from NCI-H292 cells [24]. However, the inhibition of MAPK signaling with specific pharmacological inhibitors in NCI-H292 cells had little effect on the expression of FGFBP1 induced by PLA (Supplementary Fig. S2). Here, we showed that mTORC1 signaling was activated in PLA-activated cells, as well as in OVA-induced murine asthma models. Rapamycin, an inhibitor of mTORC1, could dramatically lead to the downregulation of FGFBP1 expression and significantly reduced the number of neovascularizations in asthma models. Therefore, our current data provide the first insight into mTORC1 signaling in the expression of FGFBP1, improving our understanding of how mTORC1 activity induces angiogenesis. In addition, we revealed that rapamycin may be a potential therapeutic strategy for the treatment of angiogenesis in patients with asthma. Previous studies have illustrated that rapamycin inhibited airway remodeling through suppression of airway smooth muscle hyperplasia and hypertrophy, as well as subepithelial fibrosis in OVA- or house dust mite (HDM)-induced asthma models [54, 55]. mTOR inhibition also blocked airway inflammation and airway hyperresponsiveness (AHR) in an HDM-sensitized mouse model [56]. However, Fredriksson et al. found that in an HDM asthma model, rapamycin treatment in established asthma aggravated airway inflammation and AHR [57]. In addition, airway-specific mTOR-deficient mice exhibited more serious allergic airway inflammation and mucus hyperproduction than controls [58]. The reason for these contradictory results is unclear, but could partly be attributed to the species-specific effects of rapamycin when used in rats or mice, the different methods used for establishing asthma modeling, different concentrations of rapamycin, or other unknown differences. Further investigation is therefore needed to identify the exact reason behind these contradictory effects of rapamycin in animal models of asthma.

Various findings have demonstrated the transcriptional regulation of FGFBP1 expression [59–61]. For instance, Ray et al. proposed that the angiogenesis of colon carcinoma can be promoted by β-catenin-mediated upregulation of FGFBP1 transcription [60]. Huang et al. demonstrated that Sox12 upregulates FGFBP1 during hepatocellular carcinogenesis [62]. Zheng et al. found that KLF5 upregulation of FGFBP1 is critical for the proliferation of breast cancer cells [61]. In addition, c-Myc has been shown to promote proliferation and metastasis through directly binding to the promoter of FGFBP1 and enhancing its transcription in pancreatic adenocarcinoma [59]. c-Myc is a direct target of mTORC1, which regulates the expression of a multitude of gene products involved in cell growth and angiogenesis [63–65]. Interestingly, inhibition of c-Myc has little effect on the expression of FGFBP1 induced by PLA in NCI-H292 cells (Supplementary Fig. S3). Therefore, the possibility that mTORC1 activated FGFBP1 expression is regulated by c-Myc could be ruled out. In the current study, based on the analysis of human airway epithelial cells and OVA-induced asthma mouse models, we demonstrated that STAT3 serves as a downstream effector of mTORC1 and transcriptionally upregulates FGFBP1 through directly binding to its promoter and enhancing its transcription. In addition to human airway epithelial cells, we also illustrated that hyperactivated mTORC1 promoted the expression of FGFBP1 through activation of STAT3 in Tsc1−/− or Tsc2−/− MEFs. We also detected a positive correlation between FGFBP1 expression and p-STAT3 activity in OVA-induced asthma mouse models. Thus, STAT3-stimulated FGFBP1 transcription is a common phenomenon across species. In fact, previous studies have shown that STAT3 is activated in asthma, and its activity is associated with airway remodeling, airway inflammation, and AHR [66–68]. For example, Gavino et al. demonstrated that activated STAT3 was involved in HDM-induced airway remodeling, including increased goblet cells, epithelial cell thickness, and subepithelial smooth muscle thickness [66]. Simeone-Penney et al. found that STAT3 in the airway epithelium was required for AHR and the regulation of immune cell recruitment (including eosinophils) in an HDM-induced asthma model [68]. Thus, it is likely that activated STAT3 is involved in asthma, at least partly, by regulating transcriptional activation of FGFBP1.

To conclude, we illustrated that hyperactivation of the mTORC1-STAT3-FGFBP1 pathway in the airway epithelium contributes to the progress of angiogenesis (Fig. 8G), suggesting that this pathway should be specifically targeted for the treatment of asthma.

## Supplementary information

Supplemental Materials

Supplementary Table S1

Supplementary Table S2

Supplementary Table S3

Supplementary Figure S1

Supplementary Figure S2

Supplementary Figure S3

## Data Availability

All data generated or analyzed during this study are included in this published article and its Supplementary information files.
