# Supplementary material for: Poly-L-arginine promotes asthma angiogenesis through induction of FGFBP1 in airway epithelial cells via activation of the mTORC1-STAT3 pathway
Source: Cell Death Dis. 2021 Aug 2;12(8):761. doi: 10.1038/s41419-021-04055-2 (PMC8329163; doi:10.1038/s41419-021-04055-2)
Supplement: Supplementary file 1 — Supplemental Materials [file 41419_2021_4055_MOESM1_ESM.docx]

**Supplemental Materials**

**Supplementary Table S1.** The primer sequences used for qRT-PCR.

**Supplementary Table S2.** Clinical features and analysis of FGFBP1 expression of the subjects.

**Supplementary Table S3.** Predicted STAT3 binding sites on FGFBP1 promoter. A 2.0 kb region upstream to the transcription starting site of the FGFBP1 gene were scanned using the Jaspar transcription profile database (<http://jaspar.genereg.net>) and three putative STAT3-binding sites were predicted.

**Supplementary Figure S1.** PLA activates mTOR and STAT3 in human airway epithelial cells. NCI-H292 (**A**) and BEAS-2B (**B**) cells were treated with PLA (0, 10, 20 μg/mL) for 24 h. Cell lysates were harvested for immunoblotting with the indicated antibodies.

**Supplementary Figure S2.** PLA mediated FGFBP1 upregulation is MAPK-independent (involves p-ERK and p38 MAPK signaling). NCI-H292 cells were pretreated with PLA (20 μg/mL, 12 h) before SB203580 (30 μM, 24 h) or PD98059 (10 μM, 24 h) treatment. Cell lysates were harvested for immunoblotting with the indicated antibodies.

**Supplementary Figure S3.** Inhibition of c-Myc has little effect on the expression of FGFBP1 induced by PLA. NCI-H292 cells were pretreated with PLA (20 μg/mL, 12 h) before 10058-F4 (200 μM, 12 h) treatment. The expressions of FGFBP1 and c-Myc were detected by western blot.
