# Supplementary material for: Poly-L-arginine promotes asthma angiogenesis through induction of FGFBP1 in airway epithelial cells via activation of the mTORC1-STAT3 pathway
Source: Cell Death Dis. 2021 Aug 2;12(8):761. doi: 10.1038/s41419-021-04055-2 (PMC8329163; doi:10.1038/s41419-021-04055-2)
Supplement: Supplementary file 2 — Supplementary Table S1 [file 41419_2021_4055_MOESM2_ESM.docx]

**Supplementary Table S1.** The primer sequences used for qRT-PCR.

| Target genes | Primer sequences (5′-3′) |
| --- | --- |
| hFGFBP1-F | TCTGGGCAACACCCAGAT |
| hFGFBP1-R | GGCATGAGGTTGGATTGC |
| hPWP2-F | CCACTCGGTACAACGTCAAGT |
| hPWP2-R | TCAGGGGAGAAGGACACACTG |
| hSERPINB2-F | GCATGTTCTTGTTGCTTCCA |
| hSERPINB2-R | TTCAGCCATTTTGTCTTTGC |
| hFST-F | GGGAGAGGCCGGTGTTCCCT |
| hFST-R | TGGGGGAATACAGGGGAGCTGT |
| hFBXO34-F | CAGCAGGGAAACGCAGAGAA |
| hFBXO34-R | GATGATGCTTTACCGAGAGAGG |
| hMEX3C-F | ATGATTCGTGCATCTCGAAACA |
| hMEX3C-R | GGTCCAACCACTAATCCTACCAC |
| hGAPDH-F | CTGGGCTACACTGAGCACC |
| hGAPDH-R | AAGTGGTCGTTGAGGGCAATG |
