# Supplementary material for: Poly-L-arginine promotes asthma angiogenesis through induction of FGFBP1 in airway epithelial cells via activation of the mTORC1-STAT3 pathway
Source: Cell Death Dis. 2021 Aug 2;12(8):761. doi: 10.1038/s41419-021-04055-2 (PMC8329163; doi:10.1038/s41419-021-04055-2)
Supplement: Supplementary file 3 — Supplementary Table S2 [file 41419_2021_4055_MOESM3_ESM.docx]

**Supplementary Table S2.** Clinical features and analysis of FGFBP1 expression of the subjects

| Characteristics | | Asthma  N=15 | Healthy  N=13 | *P* value |
| --- | --- | --- | --- | --- |
| Gender | Female | 6 | 6 | 0.743 |
|  | Male | 9 | 7 |  |
| Age (years) | | 30 (21, 52) | 30 (26, 43) | 0.835 |
| BMI (Kg/m^2^) | | 23.978±2.802 | 22.022±3.421 | 0.098 |
| Eosinophils (10^9^/L) | | 0.396±0.282 | 0.126±0.091 | **0.003** |
| WBC (10^9^/L) | | 7.570±1.202 | 7.365±1.438 | 0.685 |
| Total serum IgE (KU/L) | | 225.867±107.812 | NA | - |
| FEV1 % | | 88.767±14.802 | NA | - |
| FEV1/FVC (%) | | 100.304±9.245 | NA | - |
| FeNO (ppb) | | 35 (23, 66) | NA | - |
| FGFBP1 (ng/mL) | | 65.163±11.587 | 53.672±7.296 | **0.005** |

χ² Test, Z Test and *t* Test. *BMI* body mass index, *WBC* white blood cell, *FEV1%* percentage of predicted forced expiratory volume in 1 second, *FEV1/FVC (%)* forced expiratory volume in 1 second (FEV1)/forced volume vital capacity (FVC) ratio, *FeNO* the fraction of exhaled nitric oxide, *FGFBP1* fibroblast growth factor-binding protein 1.
