# Supplementary material for: Poly-L-arginine promotes asthma angiogenesis through induction of FGFBP1 in airway epithelial cells via activation of the mTORC1-STAT3 pathway
Source: Cell Death Dis. 2021 Aug 2;12(8):761. doi: 10.1038/s41419-021-04055-2 (PMC8329163; doi:10.1038/s41419-021-04055-2)
Supplement: Supplementary file 4 — Supplementary Table S3 [file 41419_2021_4055_MOESM4_ESM.docx]

**Supplementary Table S3.** Predicted STAT3 binding sites on FGFBP1 promoter.

| Name | Score | Relative score | Start | End | Strand | Predicted sequence (5′-3′) |
| --- | --- | --- | --- | --- | --- | --- |
| R1  R2 | 9.24496  15.9841 | 0.9096  0.9912 | -752  -1500 | -742  -1490 | +  - | CTTCTGAAAAT  CTTCCCAGAAA |
| R3 | 8.25822 | 0.8976 | -1694 | -1684 | + | GTACCTGGAAA |
