# Supplementary figures and images for: Poly-L-arginine promotes asthma angiogenesis through induction of FGFBP1 in airway epithelial cells via activation of the mTORC1-STAT3 pathway
Source: Cell Death Dis. 2021 Aug 2;12(8):761. doi: 10.1038/s41419-021-04055-2 (PMC8329163; doi:10.1038/s41419-021-04055-2)

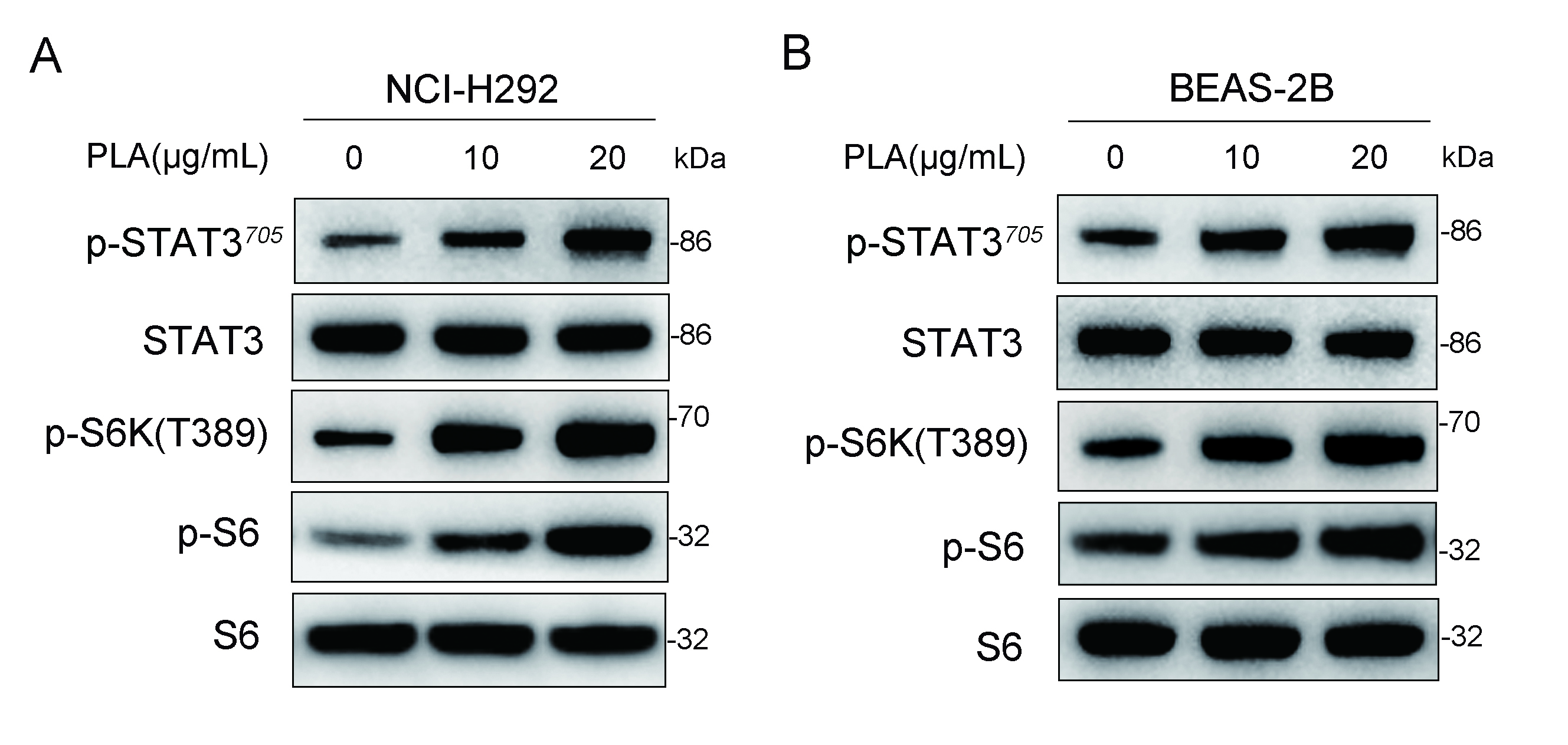

Supplement: Supplementary file 5 — Supplementary Figure S1 [file 41419_2021_4055_MOESM5_ESM.jpg]

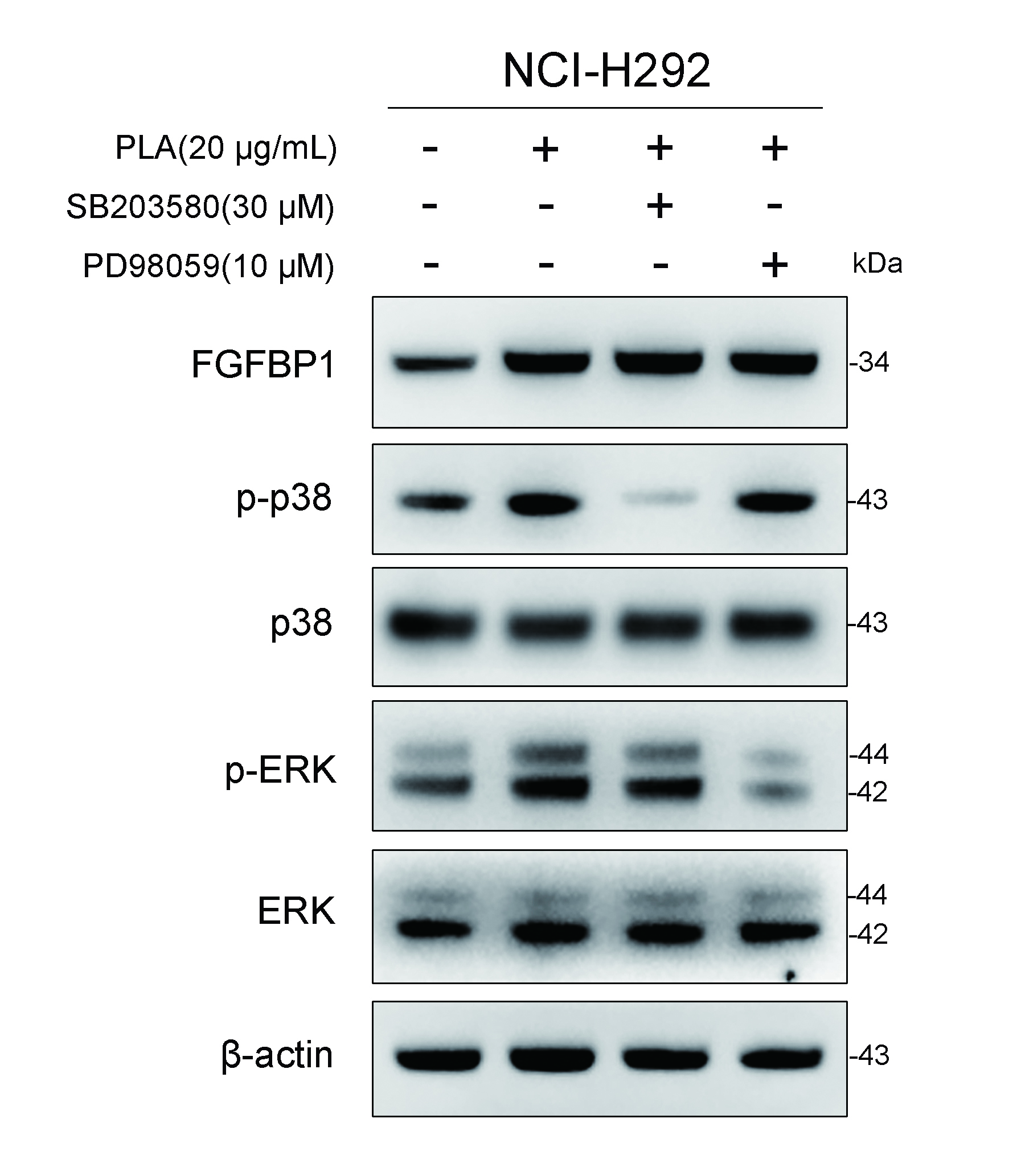

Supplement: Supplementary file 6 — Supplementary Figure S2 [file 41419_2021_4055_MOESM6_ESM.jpg]

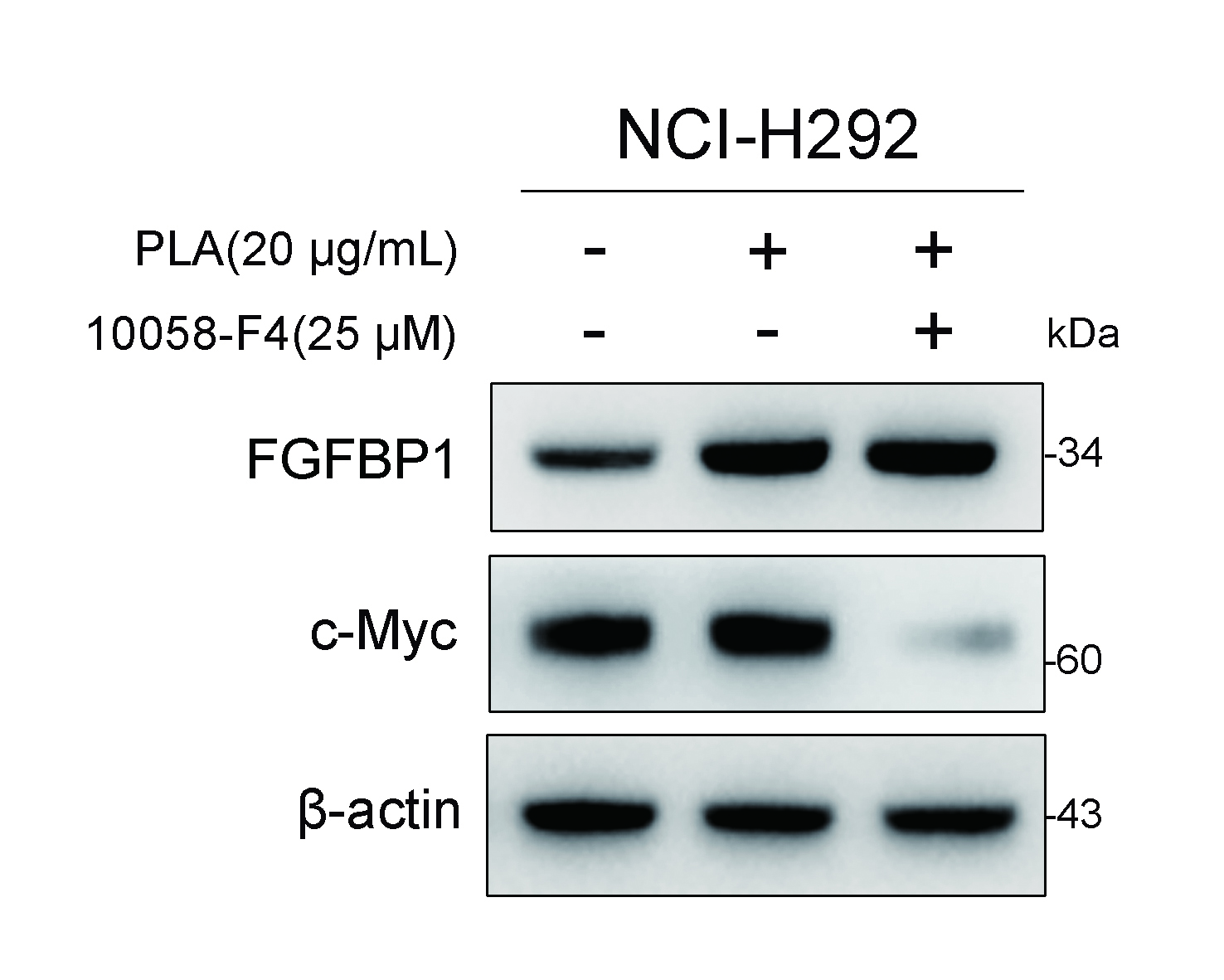

Supplement: Supplementary file 7 — Supplementary Figure S3 [file 41419_2021_4055_MOESM7_ESM.jpg]
